# Histone Deacetylase 6 Knockout Mice Exhibit Higher Susceptibility to Influenza A Virus Infection

**DOI:** 10.3390/v12070728

**Published:** 2020-07-06

**Authors:** Mark Zanin, Jennifer DeBeauchamp, Gowthami Vangala, Richard J. Webby, Matloob Husain

**Affiliations:** 1 State Key Laboratory of Respiratory Diseases, Guangzhou Medical University, 195 Dongfengxi Rd, Guangzhou 510182, China; mark.zanin@gird.cn; 2 Department of Infectious Diseases, St Jude Children’s Research Hospital, Memphis, TN 38105, USA; jennifer.debeauchamp@stjude.org (J.D.); richard.webby@stjude.org (R.J.W.); 3 Department of Microbiology and Immunology, University of Otago, Dunedin 9054, New Zealand; gowthami.vangala@otago.ac.nz

**Keywords:** influenza A virus, IAV, histone deacetylase 6, HDAC6, knockout mice, type I interferon, CD80, CXCL10, IL15, RIG-I

## Abstract

The host innate defence against influenza virus infection is an intricate system with a plethora of antiviral factors involved. We have identified host histone deacetylase 6 (HDAC6) as an anti-influenza virus factor in cultured cells. Consistent with this, we report herein that HDAC6 knockout (KO) mice are more susceptible to influenza virus A/PR/8/1934 (H1N1) infection than their wild type (WT) counterparts. The KO mice lost weight faster than the WT mice and, unlike WT mice, could not recover their original body weight. Consequently, more KO mice succumbed to infection, which corresponded with higher lung viral loads. Conversely, the expression of the critical innate antiviral response genes interferon alpha/beta, CD80, CXCL10 and IL15 was significantly downregulated in KO mouse lungs compared to WT mouse lungs. These data are consistent with the known function of HDAC6 of de-acetylating the retinoic acid inducible gene-I (RIG-I) and activating the host innate antiviral response cascade. Loss of HDAC6 thus leads to a blunted innate response and increased susceptibility of mice to influenza A virus infection.

## 1. Introduction

Influenza virus continues to be a significant burden and threat to global public health. Influenza A virus (IAV) is the prototypic and most significant member of *Orthomyxoviridae* family. IAV possesses a segmented negative-sense, single-stranded RNA genome and exhibits a broad host range. This allows IAV to constantly circulate in nature and generate genetically diverse variants through evolution [1]. These variants then cause seasonal epidemics, unpredictable pandemics and zoonotic outbreaks. Furthermore, this evolving nature of IAV has made the development of a universal vaccine difficult and increases the development of resistance against available antiviral drugs [2,3,4]. Consequently, IAV continues to cause significant morbidity and mortality, as well as productivity and economic losses worldwide annually. Considering all IAV characteristics, it is an unlikely candidate for eradication. Therefore, it is important to continue studying the molecular nature of IAV–host interactions to identify potential avenues for anti-IAV interventions.

To this end, we have discovered that host enzymes, histone deacetylases (HDACs), are anti-IAV factors. The HDACs are a family of 18 members which are classified into four classes. We and others have found that at least one member of each class, including a class II member histone deacetylase 6 (HDAC6) [5,6] exhibits antiviral properties during IAV infection [7,8,9,10,11,12]. HDAC6 is a multi-substrate deacetylase with two catalytic domains [5,6] and has been shown to exert its anti-IAV function through multiple mechanisms. This includes the downregulation of viral component trafficking to viral assembly sites on the plasma membrane [7], activation of the retinoic acid inducible gene-I (RIG-I) sensing of viral RNA [13] and the destabilisation of viral PA [14]. These findings were mostly obtained using in vitro cell culture models and their relevance during an in vivo infection has remained unclear. This prompted us to validate the anti-IAV function of HDAC6 in vivo by investigating the susceptibility of HDAC6 knockout (KO) mice to IAV infection.

## 2. Materials and Methods

### 2.1. Animals

HDAC6 KO mice germplasm [15] was received from Tso-Pang Yao (Duke University, USA) through Paul Taylor (St Jude Children’s Research Hospital, USA). The HDAC6 KO mice were reconstituted on a C57BL/6 background and bred at St Jude’s animal facility. Animal experiments were conducted with the approval of the St. Jude Children’s Research Hospital Institutional Animal Care and Use Committee (Protocol Number: 081; July 31, 2014). Mice were genotyped by standard PCR using the DNA extracted from tail tips as template and primer sets: Int-9, 5′-CTGGTTCGTCTGAAGACA-3′; Exo-10, 5′-GTGGACCAGTTAGAAGCC-3′; Zeo-1, 5′-CCATGACCGAGATCGGCGAGCA-3′ and Zeo-3, 5′-CGTGAATTCCGATCATATTCAAT-3′, flanking the targeted HDAC6 gene sequence [15].

### 2.2. Infection

Mice were inoculated intranasally with 50–450 plaque forming units (pfu) of influenza virus A/PR/8/1934 (H1N1) post-anaesthesia with isoflurane (4% in oxygen). The virus inoculum was delivered in 30 µL sterile phosphate buffered saline (PBS, pH 7.2) per mouse. Post-inoculation, mice were monitored twice daily for weight and disease symptoms (scruffy fur, hunched appearance and reduced bright-alert response), and were euthanised by CO_2_ asphyxiation and cervical dislocation if the body weight was lost by ≥30% or after 5 or 14 days of infection. The lungs were collected from mice under Avertin (2,2,2-tribromoethanol; Acros Organics, NJ, USA) anaesthesia. Lungs were homogenised and processed either to measure the virus titres as 50% tissue culture infectious doses (TCID_50_) in Madin Darby Canine Kidney (MDCK) cells according to the Reed and Muench method, Western blotting or quantitative real-time PCR.

### 2.3. Quantitative Real-Time PCR

Total RNA was isolated from the homogenised lung tissue using Nucleospin RNA isolation kit (Macherey-Nagel, Düren, Germany) and the cDNA was synthesised using PrimeScript RT reagent kit (Takara, Shiga, Japan) by following the manufacturer’s protocols. Quantitative real-time PCR was performed on the ViiA 6 real-time PCR system (Applied Biosystems, CA, USA) using the SYBR Green Select Master Mix (Life Technologies, CA, USA) and predesigned KiCqStart or custom synthesised primers obtained from Sigma-Aldrich (MO, USA). The custom primers were: beta-actin, forward, 5′-GATGTATGAAGGCTTTGGTC-3′, reverse, 5′-TGTGCACTTTTATTGGTCTC-3′; Hprt, forward, 5′-AGGGATTTGAATCACGTTTG-3′, reverse, 5′-TTTACTGGCAACATCAACAG-3′ and IFITM1, forward 5′-GAAGATGGTGGGTGATACGA-3′, reverse 5′-GCAGCGATAGACAAGGAAAC-3. The level of beta-actin or Hprt mRNA was used as a reference to normalise the levels of each target gene mRNA, and the relative change in target gene mRNA levels was calculated using ΔC_T_ method.

### 2.4. Western Blotting

Protein was extracted from the homogenised lung tissue using a lysis buffer (50 mM Tris-HCl, 150 mM NaCl, 0.5% SDS, 0.5% sodium deoxycholate, 1% Triton X-100, pH 7.4 and 1× protease inhibitor tablet (Roche, Basel, Switzerland)), and total protein concentration was determined by the Pierce™ BCA protein assay kit (ThermoFisher Scientific, MA, USA). An equal amount (50 μg) of protein from WT and KO mouse lung tissue was resolved on 8% Tris-Glycine SDS-PAGE along with SeeBlue^®^ Plus 2 protein standard (Life Technologies). The resolved polypeptides were transferred onto a Protran^®^ Premium nitrocellulose membrane (GE Healthcare, IL, USA). The membrane was blocked with 5% non-fat dry milk, and probed with the rabbit anti-HDAC6 (D21B10, Cell Signalling, MA, USA) or mouse anti-tubulin (Sigma-Aldrich) antibody, followed by the horseradish peroxidase-conjugated anti-rabbit or anti-mouse (Life Technologies) IgG. The protein bands were visualised using ECL or ECL Prime Western Blotting Systems (GE Healthcare), and the images were acquired on Odyssey Fc imaging system with Image Studio software version 5.0 (Li-COR, NE, USA) and exported as TIFF images.

### 2.5. Statistical Analysis

The statistical analyses were performed using GraphPad Prism (version 8). The *p* values were calculated using either unpaired two-tailed *t*-tests for pairwise data comparisons or two-way analysis of variance (ANOVA) incorporating Sidak’s test for multiple data set comparisons. A *p* value of ≤0.05 was considered significant.

## 3. Results

### 3.1. HDAC6 KO Mice Show Higher Weight Loss, Lower Survival and Higher Lung Virus Titres after IAV Infection

The HDAC6 gene is localised to an X chromosome [16,17] and a knockout (KO) mouse has been created [15]. We reconstituted the HDAC6 KO on a C57BL/6 background. The genotype of bred mice was determined by PCR and the expression levels of HDAC6 polypeptide, or lack thereof, in mouse lung tissue were confirmed by Western blotting (Figure S1).

**Figure 1:**
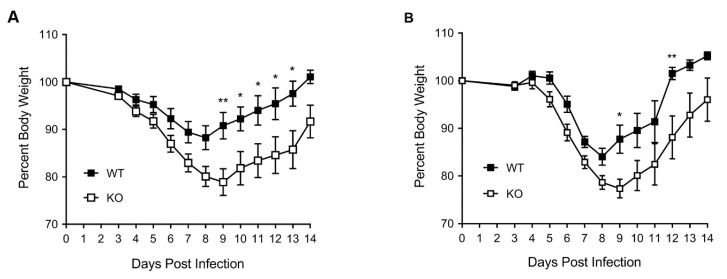
HDAC6 knockout (KO) mice exhibit higher weight loss after influenza A virus (IAV) infection. The wild type (WT) and KO male (**A**) and female (**B**) mice were inoculated intranasally with 150 pfu or 50 pfu of influenza virus A/PR/8/1934 (H1N1) (PR8), respectively. The mice were monitored daily for weight loss from 3 days post-infection until 14 days post-infection. The mouse weight on day 0 (day of inoculation) was considered 100% to determine their weight on subsequent days. The error bars represent the means ± standard errors with 95% confidence interval (male, *n* = 17 each; female, *n* = 10 each). The asterisks represent the *p* value (*, <0.05; **, <0.005) calculated by two-way ANOVA incorporating Sidak’s multiple comparisons test.

Then, 8–10-week-old, sex- and weight-matched, homozygous WT and KO littermate control mice (male and female) were inoculated intra-nasally with influenza virus A/PR/8/1934 (H1N1) (hereafter referred to as PR8), and their body weight and survival were monitored over two weeks. Both WT and KO mice started losing weight from day 4 post virus inoculation (Figure 1A). By day 8, surviving WT male mice had lost 12% of their body weight, but started recovering their weight from day 9. By day 14, WT male mice had recovered over 100% of their original weight (Figure 1A). On the other hand, the KO male mice lost weight more rapidly than WT male mice and, by day 8, they had lost a significantly higher 20% of their starting body weight (Figure 1A). Furthermore, in contrast to WT male mice, KO male mice continued to lose weight through day 9, to a significantly higher 21% (*p* = 0.0024). Nevertheless, from day 10 (*p* = 0.029), surviving KO male mice started recovering their weight albeit at a significantly lower rate compared to WT male mice. However, the KO mice could only recover 91% of their original body weight by day 14 (Figure 1A). Similarly, the WT female mice lost 16% of their body weight by day 8 and started recovering their weight from day 9. By day 14, WT female mice had recovered more than 100% of their original body weight (Figure 1B). In comparison, the KO female mice lost a significantly higher, 21% of their body weight by day 8, and continued to lose it through day 9 (23%, (*p* = 0.014)) (Figure 1B). Surviving KO female mice started recovering their body weight from day 10 and could only recover 96% of their original body weight by day 14 (Figure 1B).

**Figure 2:**
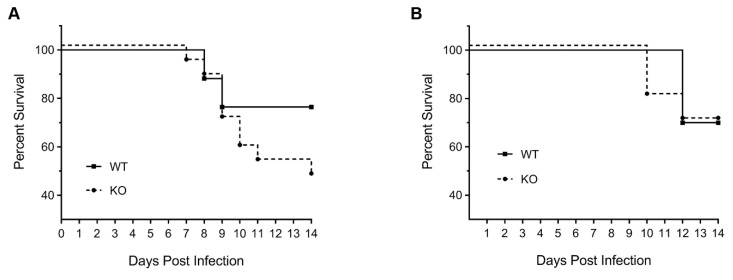
HDAC6 KO mice have lower survival rate than WT mice after IAV infection. The WT and KO male (**A**) or female (**B**) mice inoculated intranasally with 150 pfu or 50 pfu of PR8, respectively, in Figure 1 were monitored for survival due to infection over 14 days. The solid circles and squares represent the days of death (male, *n* = 17 each; female, *n* = 10 each).

Consistent with recovery in body weight, over 76% of WT male mice survived PR8 infection, whereas only 47% of KO male mice survived PR8 infection (Figure 2A). All 24% of non-surviving WT male mice were either humanely euthanised (due to losing ≥30% body weight) or naturally succumbed to infection on day 8 or 9. In contrast, the non-surviving KO male mice were either euthanised or succumbed to infection regularly during the course of the 14-day infection. The overall survival rate of WT and KO female mice after PR8 infection was similar (70%) (Figure 2B). However, 20% of KO female mice were either euthanised or succumbed to infection two days earlier (day 10) than the WT female mice (day 12).

**Figure 3:**
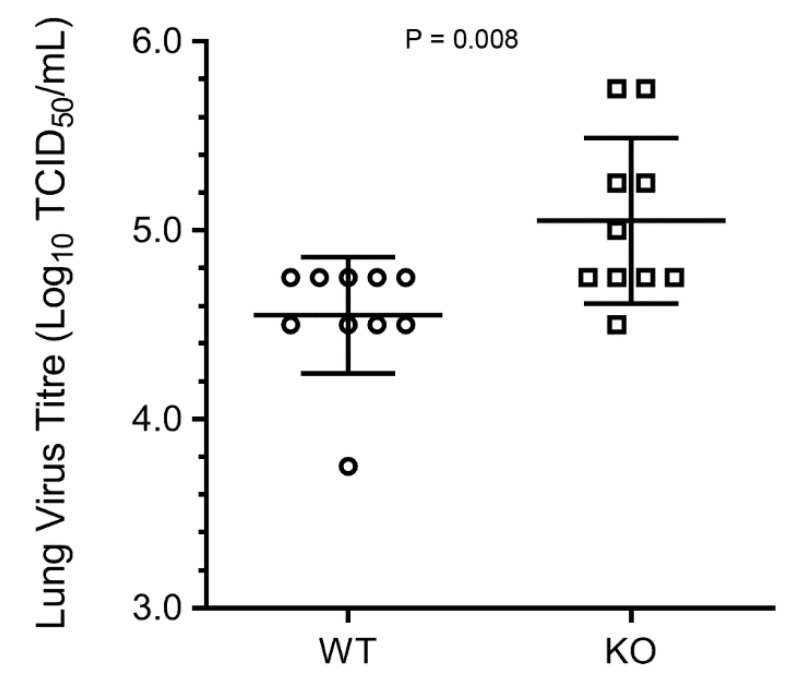
IAV progeny virus titre is higher in HDAC6 KO mouse lungs. The WT and KO male mice were inoculated intranasally with 150 pfu of PR8. After 5 days, the lungs were collected, homogenised, and virus titres were measured as 50% tissue culture infectious dose (TCID_50_) in MDCK cells according to the method of Reed and Muench. The error bars represent the means ± standard deviation with 95% confidence interval (*n* = 10 each). The *p* value was calculated by the unpaired two-tailed *t*-test.

Finally, we compared the PR8 progeny titres in the lungs of WT and KO mice. For this, WT and KO male mice were inoculated as above. After five days, the lungs were collected and processed to determine the virus titre by TCID_50_ assay. Consistent with weight loss and survival rate, the average virus titre (10^5.05^ TCID_50_/mL) in KO mouse lungs was significantly higher (*p* = 0.008) than the virus titre (10^4.55^ TCID_50_/mL) in WT mouse lungs (Figure 3).

### 3.2. The Expression of Type I Interferon and Innate Antiviral Response Genes *CD80*, *CXCL10*, and *IL15* Is Downregulated in HDAC6 KO Mouse Lungs

**Figure 4:**
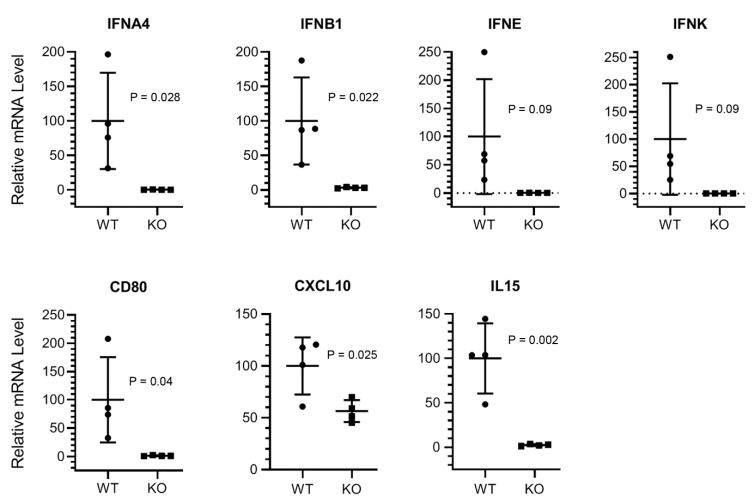
Type I interferon (IFN) and *CD80*, *CXCL10* and *IL15* genes are downregulated in HDAC6 KO mouse lungs after IAV infection. The WT and KO male mice were inoculated intranasally with 150 pfu of PR8 for 5 days. The lungs were collected and processed to detect the mRNA levels of indicated target genes and Hprt by qPCR. The qPCR was performed in triplicate, and the average mRNA level of each target gene in WT and KO mouse lungs was normalised with corresponding average Hprt mRNA level. The normalised level of each target gene mRNA in WT mouse lungs was considered 100% to determine its mRNA level in KO mouse lungs. The error bars represent the means ± standard deviation with 95% confidence interval (*n* = 4 each). The *p* value was calculated by the unpaired two-tailed *t*-test.

To gain insight into the mechanism of the higher susceptibility of KO mice to PR8 infection, we assessed the expression of type I interferon genes: interferon alpha 4 (*IFNA4*), interferon epsilon (*IFNE*); interferon kappa (*IFNK*); interferon beta 1 (*IFNB1*) and other innate antiviral response genes: C-C motif chemokine ligand 3, 4 and 5 (*CCL3*, *CCL4*, *CCL5*); cluster of differentiation 40, 80 and 86 (*CD40*, *CD80*, *CD86*); chemokine (C-X-C motif) ligand 9, 10 and 11 (*CXCL9*, *CXCL10*, *CXCL11*); gamma-interferon-inducible lysosomal thiol reductase (*IFI30*); interferon-induced protein 35, 44, 204 and 27 like 1 (*IFI35*, *IFI44*, *IFI204*, *IFI27L1*); interferon-induced protein with helicase C domain 1 (*IFIH1*); interferon-induced protein with tetratricopeptide repeats 1 and 2 (*IFIT1*, *IFIT2*); interferon-induced transmembrane protein 1 and 3 (*IFITM1*, *IFITM3*); interleukin 1 beta, 6, 12A, 15 and 18 (*IL1B*, *IL6*, *IL12A*, *IL12B*, *IL15*, *IL18*); interferon-stimulated gene 15 (*ISG15*); interferon-induced GTP-binding protein (*MX1*) and radical S-adenosyl methionine domain-containing protein 2 (*Rsad2*) in mouse lungs. The expression of many of these genes has been reported to be induced in lungs in response to IAV infection [18]. The lungs from the male WT and KO mice infected with PR8 for five days were processed for analysing the expression of either HDAC6 at polypeptide level by Western blotting (Figure S1) or the above genes at mRNA level by quantitative real-time PCR (qPCR). In addition, mRNA levels of host genes, hypoxanthine phosphoribosyl transferase (Hprt) and actin were also measured to normalise the mRNA levels of the above genes. After normalisation, the mRNA level of each gene in WT mouse lungs was considered 100% to determine its mRNA level in KO mouse lungs. We found that the average expression of four type I interferon genes—*IFNA4*, *IFNE*, *IFNK*, and *IFNB1*—was reduced by >95% in KO mouse lungs, irrespective of normalisation with Hprt (Figure 4) or actin (Figure S2). Particularly, the change in the expression level of *IFNA4* and *IFNB1* was statistically significant (Figure 4, Figure S2). Similarly, the expression of *CD80* and *IL15* was also significantly reduced (by >96%) in KO mouse lungs, irrespective of normalisation with Hprt (Figure 4) or actin (Figure S2). Furthermore, the expression of *CXCL10* in KO mouse lungs was significantly decreased by 44% when normalised to Hprt (Figure 4) and 30% when normalised to actin (Figure S2).

No consistent significance changes were observed in the expression of other innate response genes in KO mouse lungs when normalised to either Hprt (Figure S3) or actin. Nevertheless, the expression of *CCL4* was significantly decreased in KO mouse lungs by 57% (*p* = 0.028) when normalised to Hprt (Figure S3), though the difference (48%) was not significant (*p* = 0.068) when normalised to actin. Similarly, the expression of *ISG15* was significantly decreased in KO mouse lungs by 56% (*p* = 0.031) when normalised to Hprt (Figure S3), but the difference (39%) was not significant (*p* = 0.39) when normalised to actin. Conversely, the expression of *IL1B* was significantly increased in KO mouse lungs by 96% (*p* = 0.005) when normalised to actin, but was not significant (*p* = 0.32) when normalised to Hprt (Figure S3).

## 4. Discussion

Here, by using a mouse model, we were able to extend and validate our previous observation that host HDAC6 is an anti-IAV host factor [7]. We found that the HDAC6 KO mice were more susceptible to IAV infection, as they lost weight faster and were slow to recover than the WT mice. Further, the survival rate of KO mice following IAV infection was also lower. However, these changes were only significantly discernible when the mice were infected with PR8 at a dose of 150 plaque-forming units (pfu) for male and 50 pfu for female. The 50 pfu dose was too low for male mice (Figure S4). At this dose, both WT and KO mice did not start losing noticeable weight until day 6 post-virus inoculation, and all mice survived the infection and were euthanised on day 14 (Figure S4). However, the difference in weight loss pattern between WT and KO male mice was consistent with what was observed with a 150 pfu dose (Figure 1A). Specifically, the WT mice lost 8% of their original body weight by day 8, and started recovering from day 9. By day 14, all WT mice had recovered 100% of their original weight. On the other hand, the KO mice lost 12% of their body weight by day 8, and started recovering from day 9. However, by day 14, the KO mice could only recover 98% of their original body weight (Figure S4). At a higher dose (450 pfu for male, 150 and 450 pfu for female), the majority of mice in WT and/or KO groups lost ≥30% of their body weight by day 7 or 8, and were either euthanised or succumbed to infection. One of the reasons for this could be that, at a higher dose PR8 was able to effectively antagonise the HDAC6, by either facilitating the degradation of its polypeptide or downregulating its enzymatic activity in WT mice to such an extent that it resembled the KO phenotype, hence skewing the comparison of the effect of infection between WT and KO mice. Earlier, we demonstrated that PR8 infection induces the caspase-mediated cleavage of HDAC6 polypeptide and downregulates its activity in human (A549) and canine (MDCK) cells [7,19,20]. Whether this occurs in mouse cells and with other IAV strains need to be tested. The data presented here are consistent with previous findings where HDAC6 KO mice were more susceptible to infection with RNA viruses, such as vesicular stomatitis virus [13] and West Nile virus [21], Furthermore, our data are also in agreement with the finding that HDAC6-overexpressing transgenic mice were less susceptible to IAV infection [22]. Nonetheless, our data are in disagreement with Banerjee et al. [23], who showed a decrease in IAV titres in HDAC6 KO mouse lungs. However, they inoculated mice (possibly male) intra-tracheally with 50 pfu instead of intra-nasally with 150 pfu (as was done herein), and did not disclose the number of mice used to obtain these data. Furthermore, Banerjee et al. did not show or mention the HDAC6 KO mouse weight and survival in response to IAV infection. Therefore, the lack of such information prevents us from rationalising a plausible explanation for this disparity.

The significant downregulation of type I IFN genes and other important innate immune response genes, *CD80*, *CXCL10* and *IL15* in KO mouse lungs also correlated with mouse weight loss and survival rate. It is an established fact that the host IFN system is the main defence against the infection of influenza virus (and other viruses) [18]. A significantly decreased induction of some type I IFN and *CXCL10*, hence IFN-gamma genes [24] in KO mouse lungs in response to PR8 infection explains the increased susceptibility of these mice to infection. Furthermore, a significantly reduced induction of *CD80* and *IL15* genes in KO mouse lungs also indicated a compromised cytotoxic T cell- and natural killer (NK) cell-mediated antiviral response against PR8 in KO mice [25,26,27]. Although there were indications of some patterns (e.g., *CCL4*, *ISG15*; Figure S3), we could not detect a significant change in the expression of other innate antiviral response genes between WT and KO mouse lungs by qPCR. One of the reasons for this could be the timing of lung collection after infection (five days) for qPCR. Evidently, the expression kinetics of many of these genes vary and they are differentially expressed in different respiratory tract tissues in response to IAV infection [18]. A thorough (though cumbersome) time-course analysis of their expression (and viral loads) in various respiratory tract tissues—bronchus, trachea, lungs from early (e.g., one day) to late (e.g., five days) times of infection—could make those patterns significant.

Among its many functions in mammalian cells, HDAC6 also has been reported to positively regulate the activation of retinoic acid inducible gene-I (*RIG-I*) [13,21]. The RIG-I is a pathogen recognition receptor in host cell cytoplasm that recognises RNA virus infection and initiates the host innate antiviral response cascade. In addition to modifications like phosphorylation, RIG-I also undergoes acetylation, which renders RIG-I inactive in uninfected cells. The HDAC6 has been identified as a RIG-I deacetylase, and upon infection with influenza virus (and other RNA viruses, e.g., vesicular stomatitis virus, Sendai virus), the HDAC6-mediated deacetylation of RIG-I activates its RNA virus-sensing function [13,21]. Earlier, it was also reported that the HDAC6-mediated deacetylation of beta-catenin, a co-activator of interferon regulatory factor 3 (IRF3), positively regulates the IRF3 signalling in Sendai virus-infected cells [28].

In conclusion, the data presented here strengthen the claim that HDAC6 is an important component of host defence against the influenza virus infection. Further studies are warranted to elucidate the role of HDACs and their counterpart, HATs (histone acetyltransferases), both central regulators of host acetylation machinery, during the influenza virus infection, and to determine their potential as targets for anti-influenza interventions.

## Supplementary Materials

The following are available online at https://www.mdpi.com/1999-4915/12/7/728/s1, Figure S1: Confirmation of HDAC6 knockour mouse genotype and phenotype; Figure S2: Type I interferon (IFN) and *CD80*, *CXCL10* and *IL15* genes are downregulated in HDAC6 KO mouse lungs after influenza (IAV) infection; Figure S3: Relative expression of innate immune response genes in WT and KO mouse lungs after IAV infection; Figure S4: Percent body weight of WT and KO male mice inoculated with 50 pfu dose of IAV.
