# Supplementary material for: Histone Deacetylase 6 Knockout Mice Exhibit Higher Susceptibility to Influenza A Virus Infection
Source: Viruses. 2020 Jul 6;12(7):728. doi: 10.3390/v12070728 (PMC7411611; doi:10.3390/v12070728)
Supplement: Supplementary file 1 [file viruses-12-00728-s001.pdf]

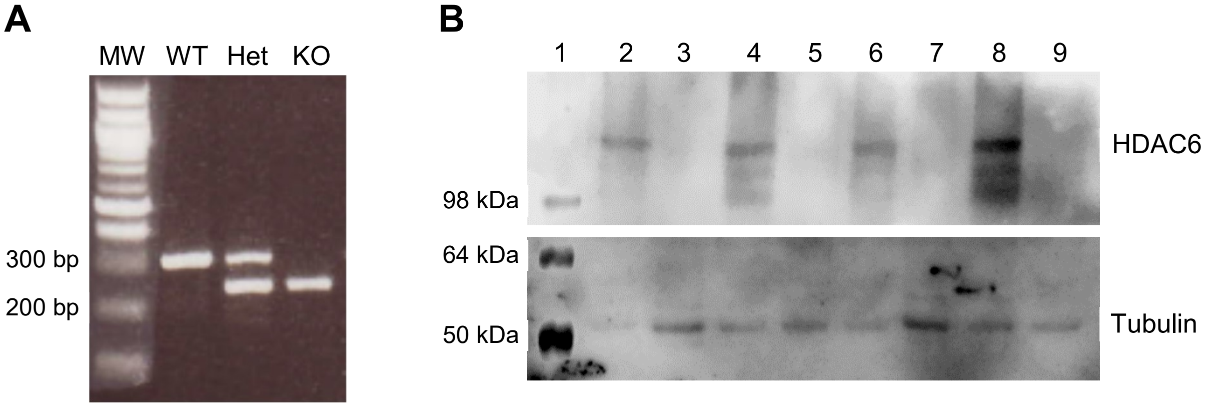

Figure S1. Confirmation of HDAC6 knockout mouse genotype and phenotype. (A) The DNA extracted from mouse tail tips was used as a template to perform standard PCR using primers flanking the targeted HDAC6 gene sequence. The PCR products were resolved on agarose gel to differentiate the wild type (WT), heterozygous (Het) and knockout (KO) alleles. MW, molecular weight; bp, base pair. (B) Protein extracted from 4 WT (lanes 2, 4, 6 and 8) and 4 KO (lanes 3, 5, 7 and 9) mouse lungs was resolved on SDS-PAGE, and HDAC6 and tubulin polypeptides were detected by western blotting. Lane 1, molecular weight; kDa, kilo Dalton.

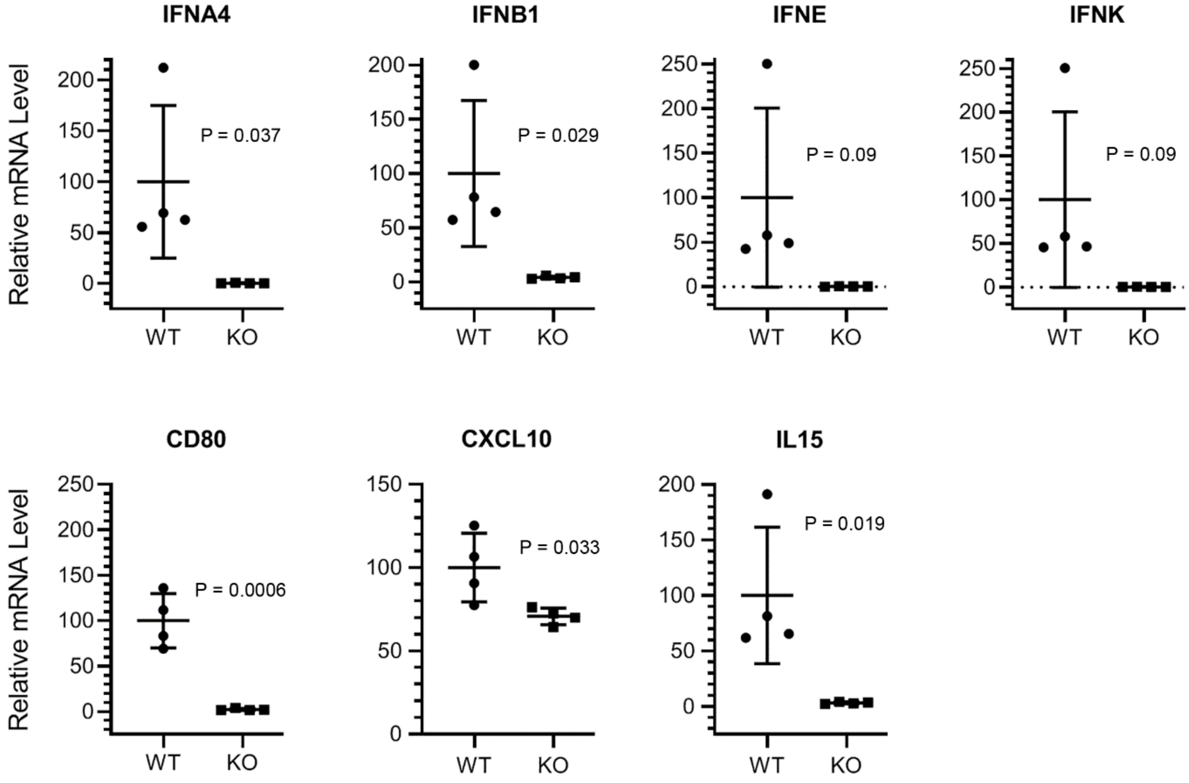

Figure S2. Type I interferon (IFN) and *CD80*, *CXCL10* and *IL15* genes are downregulated in HDAC6 KO mouse lungs after influenza A virus (IAV) infection. The WT and KO male mice were inoculated intranasally with 150 pfu of influenza virus A/PR/8/1934 (H1N1) (PR8) for 5 days. The lungs were collected and processed to detect the mRNA levels of indicated target genes and actin by quantitative real-time PCR (qPCR). The qPCR was performed in triplicates and the average mRNA level of each target gene in WT and KO mouse lungs was normalised with corresponding average actin mRNA level. The normalised level of each target gene mRNA in WT mouse lungs was considered 100% to determine its mRNA level in KO mouse lungs. The error bars represent the means ± standard deviation with 95% confidence interval (n = 4 each). The p value was calculated by the unpaired two-tailed t-test.

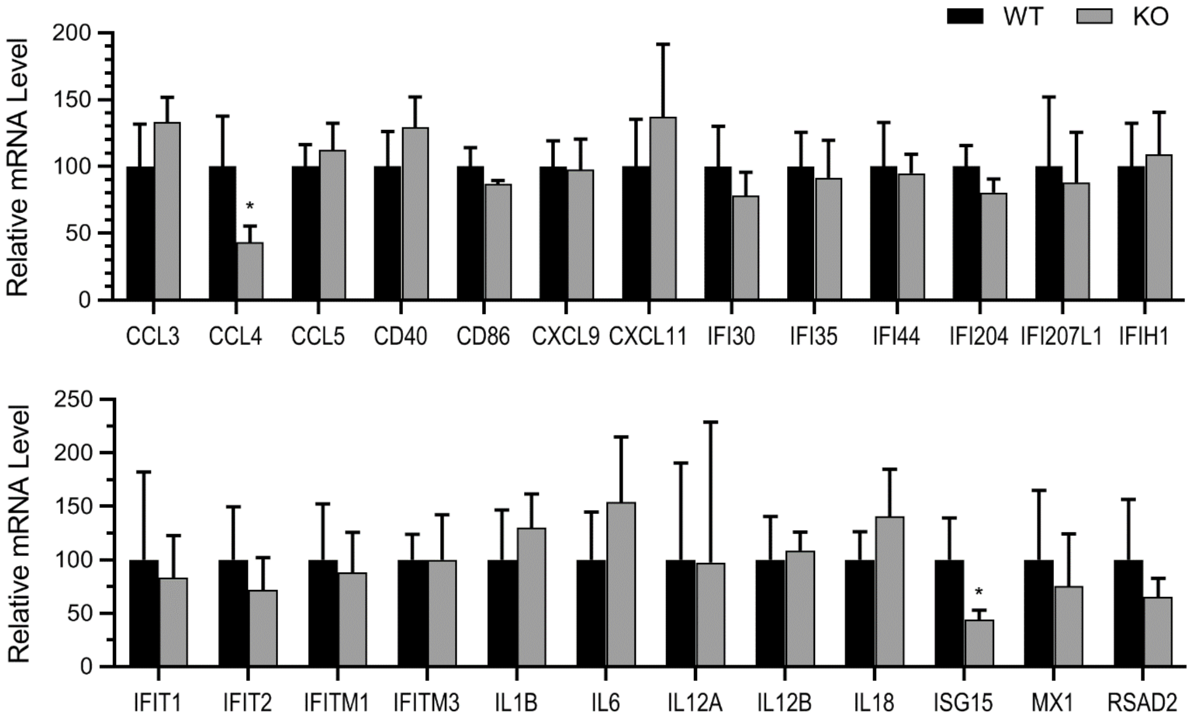

Figure S3. Relative expression of innate immune response genes in WT and KO mouse lungs after IAV infection. The WT and KO male mice were inoculated intranasally with 150 pfu of PR8 for 5 days. The lungs were collected and processed to detect the mRNA levels of indicated target genes and Hprt by qPCR. The qPCR was performed in triplicates and the average mRNA level of each target gene in WT and KO mouse lungs was normalised with corresponding average Hprt mRNA level. The normalised level of each target gene mRNA in WT mouse lungs was considered 100% to determine its mRNA level in KO mouse lungs. The error bars represent the means ± standard deviation with 95% confidence interval ($n = 4$ each). The asterisks represent the p value (CCL4, 0.028; ISG15, 0.031) calculated by the unpaired two-tailed t-test.

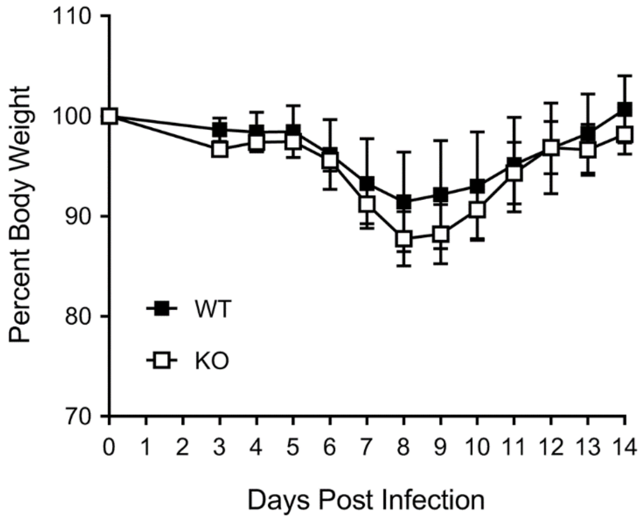

Figure S4. Percent body weight of WT and KO male mice inoculated with 50 pfu dose of IAV. The mice were inoculated intranasally with PR8, and monitored daily for weight loss from 3 days post infection till 14 days post-infection. The mouse weight on day 0 (day of inoculation) was considered 100% to determine their weight on subsequent days. The error bars represent the means ± standard errors with 95% confidence interval (n = 6 each).
